# A Lead Field Two-Domain Model for Longitudinal Neural Tracts—Analytical Framework and Implications for Signal Bandwidth

**DOI:** 10.1155/2020/5436807

**Published:** 2020-05-29

**Authors:** G. Fischer, M. Kofler, M. Handler, D. Baumgarten

**Affiliations:** ^1^Institute of Electrical and Biomedical Engineering, UMIT-Private University for Health Sciences, Medical Informatics and Technology, Eduard-Wallnoefer-Zentrum 1, 6060 Hall in Tirol, Austria; ^2^Department of Neurology, Hochzirl Hospital, Zirl, Austria; ^3^Institute of Biomedical Engineering and Informatics, Technische Universitaet Ilmenau, Ilmenau, Germany

## Abstract

Somatosensory evoked potentials are a well-established tool for assessing volley conduction in afferent neural pathways. However, from a clinical perspective, recording of spinal signals is still a demanding task due to the low amplitudes compared to relevant noise sources. Computer modeling is a powerful tool for gaining insight into signal genesis and, thus, for promoting future innovations in signal extraction. However, due to the complex structure of neural pathways, modeling is computationally demanding. We present a theoretical framework which allows computing the electric potential generated by a single axon in a body surface lead by the convolution of the neural lead field function with a propagating action potential term. The signal generated by a large cohort of axons was obtained by convoluting a single axonal signal with the statistical distribution of temporal dispersion of individual axonal signals. For establishing the framework, analysis was based on an analytical model. Our approach was further adopted for a numerical computation of body surface neuropotentials employing the lead field theory. Double convolution allowed straightforward analysis in the frequency domain. The highest frequency components occurred at the cellular membrane. A bandpass type spectral shape and a peak frequency of 1800 Hz was observed. The volume conductor transmitting the signal to the recording lead acted as an additional bandpass reducing the axonal peak frequency from 200 Hz to 500 Hz. The superposition of temporally dispersed axonal signals acted as an additional low-pass filter further reducing the compound action potential peak frequency from 90 Hz to 170 Hz. Our results suggest that the bandwidth of spinal evoked potentials might be narrower than the bandwidth requested by current clinical guidelines. The present findings will allow the optimization of noise suppression. Furthermore, our theoretical framework allows the adaptation in numerical methods and application in anatomically realistic geometries in future studies.

## 1. Introduction

Somatosensory evoked potentials (SEPs) are a well-established tool for studying the conduction of stimulated peripheral activity in sensory pathways to the somatosensory cortex in clinical routine [1–3]. They allow the evaluation of volley conduction along the entire sensory pathway. For example, signals evoked by a stimulus applied to the tibial nerve at the ankle may be obtained at the popliteal fossa, over the sacral, lumbar, thoracic, and cervical spinal cord and over cortical regions. An early standard for performing such recordings is defined in [4] and provides the basis for routine recording up to now.

While in [4], bandwidth recommendations go up to 3 kHz, more recent experimental studies on high-frequency components of neural signals focus on a more narrow range of only several hundreds of hertz [5–7]. Most of these studies investigated intracortical data. As high-frequency components appear damped in a lead which is distant from the source, only a few studies exist reliably detecting components in a high-frequency band on the body surface [8].

Besides experimental data, modeling can be used for predicting spectral properties of bioelectric signals. In particular, in cardiac electrophysiology, model-based spectral analysis is a well-established tool [9, 10], and recent studies show impressive correlation of simulated and experimental data even for relatively small atrial signal components [11].

Clark and Plonsey [12] developed an early analytical model for computing the extracellular potential of a single axon. Their model was based on a partial differential equation (PDE). However, due to the complex microanatomical structure of nerves or neural tracts consisting of thousands of axons organized in fascicles, PDE-based models are computationally demanding. They require modeling of each single fiber. Some recent studies have applied PDE models for studying stimulation of peripheral nerves by electrodes [13, 14]. However, for studying stimulation, only relatively short time intervals (significantly less than 1) need to be considered. For modeling lead field evoked neural activity (i.e., activity recorded by a lead located distantly from the sources of the electric field), much longer time intervals (significantly more than 10) are required rendering PDE approaches computationally unattractive.

In this study, we present a novel theoretical framework using distributed dipoles along a central axis of a nerve, fascile, or neural tract for efficiently computing the lead field generated by such a structure. Thus, a tool is provided, which allows for multiscale modeling by linking cell models with body surface potentials. Our approach allows using statistical distribution functions for computing the potential field generated by a large number of axons. Furthermore, the use of Fourier transform (FT) allows a straightforward investigation of spectral signal properties.

We aimed at developing our theoretical framework for analytic expressions and, therefore, chose a simplified half-space geometry which approximates longitudinal conduction of a volley within a spinal tract. An early study revealed low-amplitude (<1*μV*) traveling waves generated by longitudinal spinal volley conduction, which can be observed essentially along the thoracic compartment of the spine [15]. It must not be confused with stationary synaptic spinal signals in the lumbosacral region (generated within the spinal cord gray matter) and in the cervical region (generated by subcortical structures such as the brain stem). We will discuss the generalization of our approach to other longitudinal neural structures at the end of the manuscript.

## 2. Methods

In this section, we present the key features of our analytical framework. All detailed mathematical derivations are listed in the Appendices (available here) which are provided as an online supplement.

### 2.1. Lead Field Two-Domain Model—Concept

**Figure 1:**
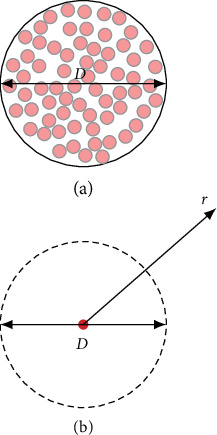
Concept of the lead field two-domain model. (a) A neural tract of diameter *D* contains a large number of individual fibers (light red) surrounded by myelin layers (gray). (b) For computation of a lead field potential in the distant field point *r*, the individual fibers can be considered on a central axis of the neural tract (red, active domain). The current return in the volume conductor of average bulk conductivity *κ* generates the extracellular potential field *ϕ*.

The concept underlying the lead field two-domain model is depicted in Figure 1. A neural tract contains several hundreds or thousands of axons. For a volley, action potentials traveling along the tract activate individual axons with a significant temporal dispersion. Here, significant dispersion means that the variation in the onset of activation may be larger than the duration of a membrane action potential pulse (de- and repolarization) and individual axons may fire even more than one pulse.

We aimed at computing the electric potential in the lead field of a neural tract. Here, the distance *r* of a field point (i.e., a lead) from the tract is significantly larger than a typical diameter *D* of the tract. At a sufficiently large distance *r*, the individual location of each axon inside the tract becomes negligible. For computing a lead field potential, we assumed that all axons are located on a central axis of the tract. A membrane action potential traveling along individual axons drives intracellular currents. Typically, action potentials are conducted with a speed in the order of 40 ms^−1^ to 80 ms^−1^ and have a duration of 0.5 ms to 1.0 ms. Thus, the activated segment of each axon has a length of some centimeters. Note that this is much larger than the diameter *d* of an individual axon, larger than the distance between adjacent nodes of Ranvier, and larger than the diameter *D* of the entire tract. Therefore, we neglected anatomical microstructures such as myelin layers and assumed an average bulk conductivity *κ* for the extracellular current return pathway.

Thus, we considered two domains: an active intracellular domain and a passive extracellular or bulk domain. In the first step, we computed the signal generated by a single axon. In the second step, we applied superposition of dispersed axonal signals for obtaining the compound action potential (CAP) of a neural tract.

### 2.2. Axonal Dipoles Model

**Figure 2:**
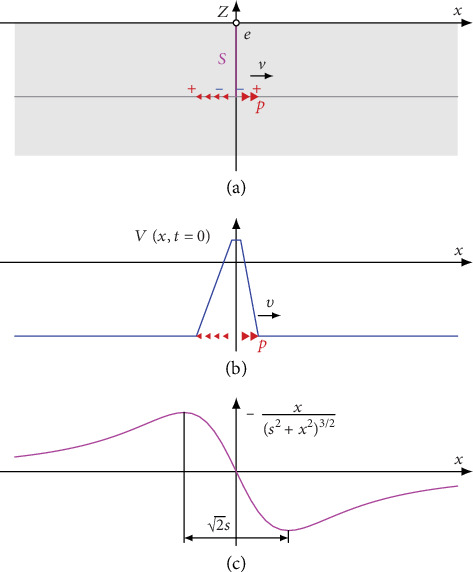
(a) Geometry of the model. The axon is located at a distance *s* from the body surface. The observation electrode *e* is placed at the origin. (b) Membrane potential *V* along the axon. At *t* = 0 the peak of *V* is located at *x* = 0. The membrane potential propagates with a velocity *v* in the *x* direction. Membrane potential gradients generate distributed moving dipole sources *p*. They are shown in (b) and (a). Due to the opposite orientation of de- and repolarization, a quadrupole source pattern is obtained. (c) The lead field is an odd function with a single oscillation. The distance between the two extrema is $\sqrt{2}s$.

We considered the signal generated by a single axon located at the central axis of a neural tract.  Figure 2 gives an overview on the axonal dipole model. Geometrically, the axon is located within a conducting half-space parallel to the body surface at the depth *s*. Along the axon (*x* direction), the membrane action potential *V*(*x*, *t*) propagates with a velocity *v*. This generates spatial gradients *dV*/*dx* driving impressed intracellular currents. We interpreted these source currents as a distribution of moving dipoles *p* creating an electric potential *ϕ*_*A*_ in the surrounding tissue and at the body surface. The body surface potential *ϕ*_*U*_ created by a unit dipole at the observation electrode *e* (located at the origin) was obtained by

(1) $$\begin{array}{c} \phi_{U}\left(x\right)=-\frac{1}{2\pi \kappa }\frac{x}{{\left(s^{2}+x^{2}\right)}^{3/2}}. \end{array}$$

Dipole strength is proportional to the first temporal derivative of the membrane potential (see Appendix A.1). We translated the movement of the membrane action potential along the axon into a convolution term and obtained the body surface potential *ϕ*_*A*_ created by the axon

(2) $$\begin{array}{c} \phi_{A}\left(t\right)=-\frac{d^{2}}{8}\frac{\sigma }{\kappa }\int_{-\infty }^{\infty }\frac{dV\left(t-\tau \right)}{dt}\frac{v\tau }{{\left(s^{2}+v^{2}\tau^{2}\right)}^{3/2}}d\tau . \end{array}$$

Here, *σ* is the conductivity of the intracellular space inside the axon and *d* is the axon diameter. For integrating all dipoles along the axon dimension *x*, we introduced the auxiliary time shift *τ* and replaced *x* by *vτ*.

Applying convolution theory and time continuous Fourier transform (FT), the frequency spectrum of the axonal signal was obtained by the product of the FT of two terms. The first (source) term is the temporal derivative of the membrane potential. The second (volume conductor) term reflects the lead field of a moving dipole source

(3) $$\begin{array}{c} F\left\{\phi_{A}\left(t\right)\right\}=-\frac{d^{2}}{8}\frac{\sigma }{\kappa }F\left\{\frac{dV\left(t-\tau \right)}{dt}\right\}F\left\{\frac{vt}{{\left(s^{2}+v^{2}t^{2}\right)}^{3/2}}\right\}. \end{array}$$

### 2.3. Neural Pathway CAP

A neural pathway is formed by a large number of *N* axons. For a volley traveling along this pathway, activation displays a temporal dispersion *T* in individual axons. This relates to a time shift of action potentials between axons. For enabling an analytical treatment, we first assumed equal diameter *d* and equal conduction velocity *v* in all axons. The remaining variation in axonal activity is modeled by a statistical distribution of activation dispersion *δ*(*T*).

The spectrum of the CAP was obtained by multiplying the spectrum of a single axon with the spectrum of the statistical distribution of the dispersion *δ*(*T*) (see Appendix B)

(4) $$\begin{array}{c} F\left\{\phi_{N}\left(t\right)\right\}=\sqrt{2\pi }F\left\{\phi_{A}\left(t\right)\right\}F\left\{\delta \left(T\right)\right\}. \end{array}$$

In the time domain, the CAP was obtained by a convolution of the signal of a single axon *ϕ*_*A*_ with the distribution of time shift *δ*(*T*). Assuming a normal distribution of time shifts *T* (standard deviation *τ*), we obtained in the frequency domain (see Appendix B)

(5) $$\begin{array}{c} F\left\{\delta \left(T\right)\right\}=\frac{N}{\sqrt{2\pi }}\exp\left(-2\pi^{2}f^{2}\tau^{2}\right). \end{array}$$

The spectrum of the distribution function (5) has a maximum at *f* = 0 (DC) and continuously converges to zero with increasing frequency, acting as a low-pass component. This holds true also for any other statistical distribution of the dispersion. According to basic theory, the Fourier transform of a probability density function *δ*(*T*) is called the characteristic function of the distribution. In Appendix B.1, we summarize some basic theorems on characteristic functions [16], proving the low-pass characteristic for any distribution. Appendix B.2 depicts examples of characteristic distribution functions in order to exemplify the described low-pass filter effect.

In the time domain, the CAP signal *ϕ*_*N*_ was obtained by inverse Fourier transform of (4).

### 2.4. Model Analysis

We observed that our analytical model represents the frequency spectrum of the lead field potential *ϕ*_*A*_ by the product of three different terms:

- The membrane potential derivative (source term, obtained from a cell model)
- The lead field (moving dipoles)
- The dispersion term

**Figure 3:**
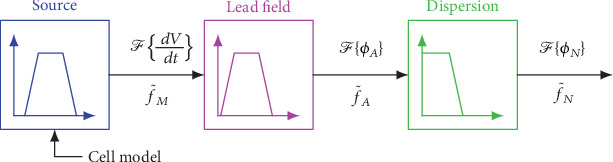
Signal generation model in the frequency domain (see text).

Figure 3 displays this observation schematically. We analyzed the properties of each of the three components and their interaction.

**Figure 4:**
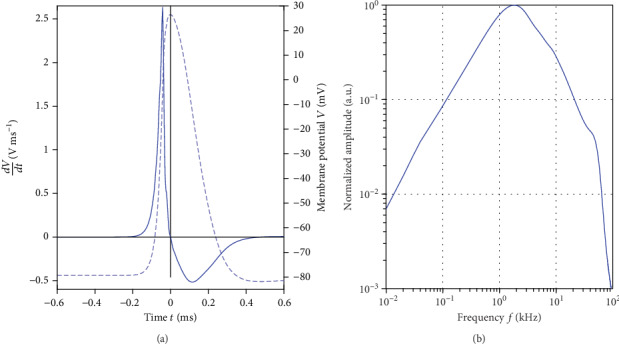
(a) Membrane action potential (dashed) of a sensory axon [14] and its temporal derivative (solid). For each trace an individual scale was applied on the ordinate. Time basis was chosen such that the potential maximum is at *t* = 0. (b) Normalized absolute amplitude of the frequency spectrum of the action potential derivative in a double logarithmic plot. The maximum was observed at 1.83 kHz. The amplitude converged to zero for low and high frequencies.

*Source term.* Figure 4 exemplarily depicts the action potential of a sensory axon [14] together with its first temporal derivative and the frequency spectrum of the derivative. The direct current content in the spectrum is zero as the membrane potential always starts at resting potential before activation, and it returns again to resting potential after activation.

Furthermore, the derivative of an action potential traveling along the axon is a piecewise continuous function. Thus, we concluded from basic theory of Fourier transform that the spectrum approaches zero also with increasing frequency (see Figure 4 and Appendix B). Thus, the frequency spectrum of the source term is of “bandpass type.” We obtained a coarse estimate ${\tilde{f}}_{M}$ of the peak frequency in the spectrum of the derivative by taking the inverse of the time interval of membrane activation Δ*T*_*M*_ (i.e., the duration of an activation cycle from the beginning of depolarization to the end of repolarization)

(6) $$\begin{array}{c} {\tilde{f}}_{M}=\frac{1}{\Delta T_{M}}. \end{array}$$

*Lead field.* The lead field transmits the source term to the potential *ϕ*_*A*_ at the observation electrode *e*. The lead field is an odd function (see Figure 2). Thus, the integral under the function yields zero, and again, there is no DC content in the spectrum. Furthermore, the volume conductor term is continuous. Again, the amplitude spectrum approaches zero with increasing frequency. Thus, also the frequency spectrum of the lead field term is of “bandpass type.”

The lead field term yields a function with a single oscillation and point-symmetric shape (see Figure 2). For coarsely estimating a center frequency ${\tilde{f}}_{L}$ of this peak, we considered the distance between the two extrema of the lead field ($\sqrt{2}s$). Within this interval, half oscillation occurs. Thus, the center frequency ${\tilde{f}}_{L}$ was estimated by taking the inverse of twice the time a dipole needs for passing the distance between the two extrema

(7) $$\begin{array}{c} {\tilde{f}}_{L}=\frac{1}{2\Delta T_{L}}\;\cdots \;\Delta T_{L}=\frac{\sqrt{2}s}{v}. \end{array}$$

Here, ${\tilde{f}}_{L}$ was a property of a model component, not of the signal. For coarsely estimating a center frequency of the axonal signal ${\tilde{f}}_{A}$ (i.e., the signal at the output of the moving dipole component in Figure 3), we considered also the duration of an activation cycle Δ*T*_*M*_:

(8) $$\begin{array}{c} {\tilde{f}}_{A}=\frac{1}{\Delta T_{M}+2\Delta T_{L}}. \end{array}$$

*Dispersion.* For obtaining a coarse estimate of the center frequency ${\tilde{f}}_{N}$ of a lead field compound action potential *ϕ*_*N*_ of a neural pathway, we considered that temporal dispersion increases the duration of the CAP signal with respect to the duration of an axonal signal. The width of the dispersion pulse was estimated by 4*τ* as about 95% of the individual activation pulses are within the interval of ±2*τ* from the center of the normal distribution [17].

(9) $$\begin{array}{c} {\tilde{f}}_{N}=\frac{1}{\Delta T_{M}+2\Delta T_{L}+4\tau }. \end{array}$$

Only four biophysically relevant parameters enter this estimation: duration of an activation cycle Δ*T*_*M*_, conduction velocity *v*, depth *s*, and standard deviation of dispersion *τ*.

### 2.5. Simulations

**Figure 5:**
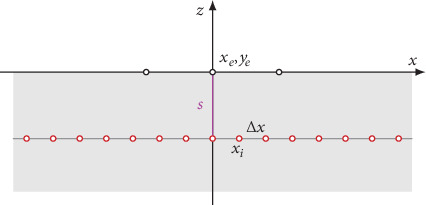
Schematic drawing of the discrete model underlying the numerical approximation. At the body surface, the potential *ϕ*_*A*_ is calculated in *E* electrodes (black circles, coordinates *x*_*e*_, *y*_*e*_). The axon is subdivided into *I* source points *x*_*i*_ at spacing Δ*x* (red circles).

For numerical evaluation of axonal potentials (2), we replaced integration by discrete summation. In Appendix A.2, we describe this in detail and generalize our concept to multiple observational electrodes or leads located at the coordinates (*x*_*e*_, *y*_*e*_) on the body surface. The discrete model is depicted in Figure 5. As outlined in Appendix A.2 a linear equation was established for computing the discrete axonal potentials

(10) $$\begin{array}{c} \Phi_{A}=LD. \end{array}$$

Here **L** is the lead field matrix relating the source points to the leads and **D** is the dipole matrix holding the primary source terms. The propagation of the membrane action potential at constant speed *v* (schematically depicted in Figure 2) causes a systematic spatial displacement of the source dipoles in each row (i.e., at each time step) of **D**. This is equivalent to the convolution in the temporal domain.

We simulated the action potential of a sensory axon applying the ionic current model described in [14] by using the software NEURON (http://www.neuron.yale.edu/neuron/). For minimizing numerical effects in our analysis, we selected fine grids for discretization. According to the analysis in Section 2.4, we expected highest frequencies at the cell membrane. Therefore, we chose the smallest time step 2 *µ*s for investigating the properties of the membrane action potential. For simulating body surface potentials, we increased the time step to 5 *µ*s. This yielded a Nyquist-frequency of 100 kHZ which is sufficient for accurately investigating spectral features of the body surface potential up to several kHz. We linked spatial discretization to the chosen time step by selecting Δ*x* = *v*Δ*t*. We simulated all signals in a time interval of -100 ms to 100 ms. We applied time discrete FT for computing all spectra obtaining a frequency resolution of 5 Hz.

In the first step, we varied the axon diameter *d* in three steps (7 *µ*m, 10 *µ*m, and 13 *µ*m) in the NEURON model for investigating the effect on the membrane action potential and conduction velocity. For studying the dependency of results from physiologic variation, we assigned three different values for the depth *s* of the neural pathway (35 mm, 50 mm, and 65 mm) [18, 19] and for the conduction velocity *v* (45 ms^−1^, 60 ms^−1^, and 75 ms^−1^) [14, 15].

According to equation (2), the amplitude of the axonal potential depends on the ratio of the intracellular conductivity to the bulk conductivity *σ*/*κ*. The intracellular conductivity is the inverse of the cytoplasmic resistivity. We used the same value *σ* = 1.4 Sm^−1^ as it was used for the NEURON model of sensory axons [14, 20] at body temperature. Bulk conductivity is significantly smaller and varies considerably between tissue types [14, 21]. Thus, we assumed a mean ratio *σ*/*κ* = 8. In order to account for the uncertainty in this ratio, we doubled and halved this value in our computations.

For all combinations of parameters, we computed the axonal potential *Φ*_*A*_. For illustrating the spatial field distribution, we simulated the potential for 10 *µ*m axon diameter at 50 mm depth within a square of 200 mm edge length centered at the origin (2.5 mm grid spacing).

For simulating neural potentials, we assigned three values to the standard deviation *τ* of temporal dispersion (1.0 ms, 1.25 ms, and 1.5 ms) and assumed the largest dispersion at the slowest conduction velocity. We chose an axon diameter of 10 *µ*m [14, 22] and assumed that *N* = 2000 axons define a neural pathway.

## 3. Results

*Source term.* For the three axon diameters (7 *µ*m, 10 *µ*m, and 13 *µ*m), the NEURON model delivered a conduction velocity of 43.6 ms^−1^, 60.0 ms^−1^, and 75.1 ms^−1^, respectively. Variation in diameter had only a minor effect on the action potential. For all three simulations, peak-to-peak amplitude (maximum at activation vs. minimum during hyperpolarization) was in the range of 107.9 mV to 108.8 mV. The time span between the two potential extrema was in the range of 480 *µ*s to 482 *µ*s. Time for activation (i.e., from crossing of the -70 mV level to the maximum) was in the range of 92 *µ*s to 94 *µ*s. Thus, we obtained a pulse duration (de- and repolarization) of Δ*T*_*M*_ = 0.57 ms yielding ${\tilde{f}}_{M}=1750$ Hz. We arbitrarily assigned *t* = 0 to the maximum of the membrane action potential. As predicted by our model analysis, the highest frequency in the signal generation chain was observed at the cell membrane (derivative of the action potential). Here, we obtained a bandpass type frequency spectrum with significant signal components in the kHz range (see Table 1 and Figure 4).

*Lead field.* For the three axon diameters (7 *µ*m, 10 *µ*m, and 13 *µ*m), we obtained peak-to-peak amplitudes (global maximum vs. minimum) of 0.44 nV, 1.24 nV, and 2.61 nV, respectively, at *σ*/*κ* = 8 and a depth of 50 mm. Taking into account also the uncertainty in conductivity and variations in depth, body surface peak-to-peak amplitude generated by single spinal cord fibers may vary in the range of approximately 0.1 nV to 10 nV. The peak frequencies in the frequency spectrum of the three axonal potentials were 215 Hz, 300 Hz, and 315 Hz, respectively. Peak frequency increased with increasing diameter due to the increase in conduction velocity.

**Figure 6:**
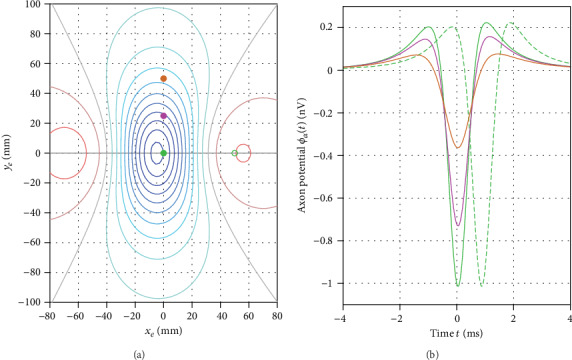
(a) Axonal potential *ϕ*_*A*_ generated by a single axon of 10 *µ*m diameter at a depth of 50. The conductivity ratio was set to *σ*/*κ* = 8. Isopotential lines are shown in steps of 0.1 nV. Circles indicate the location of the four observation leads which are depicted in (b). (b) The green solid trace depicts the axonal potential *ϕ*_*A*_ at the origin. Shifting the observation lead by 50 mm along the axon (green dashed line) introduced a time shift of 0.83 ms while preserving signal morphology. Lead displacements in a direction perpendicular to the axon are indicated by a magenta trace (*y*_*e*_ = 25 mm) and an orange trace (*y*_*e*_ = 50 mm). With increasing distance from the axon amplitude decreased (peak-to-peak amplitude 1.24 nV, 0.89 nV, and 0.44 nV). Furthermore, the shape of the negative peak broadened which was also reflected by a decreasing peak frequency (300 Hz, 265 Hz, and 210 Hz).

The potentials computed for an axon diameter of 10 *µ*m at a rectangular grid are depicted in Figure 6. The field pattern reflects the underlying quadrupole source. When displacing an observation point along the axon, the signal displayed a temporal offset, while its morphology was preserved. In the rectangular direction, amplitude decreased with increasing distance from the source, as did the peak frequency. In the spatial and temporal domain, activation of the axon underneath an observation point was reflected by a potential minimum. Due to the asymmetric shape of the action potential, the minimum was slightly shifted away from *t* = 0. However, this effect amounted to only 0.06 ms. Two small positive peaks were observed, before and after the activation minimum, respectively. This signal morphology was caused by the quadrupole source pattern shown in Figure 2. The morphology was slightly asymmetric with the second maximum being slightly larger than the first. The difference in the amplitude of the positive peaks was mainly caused by the small hyperpolarization wave (about 2 mV).

**Figure 7:**
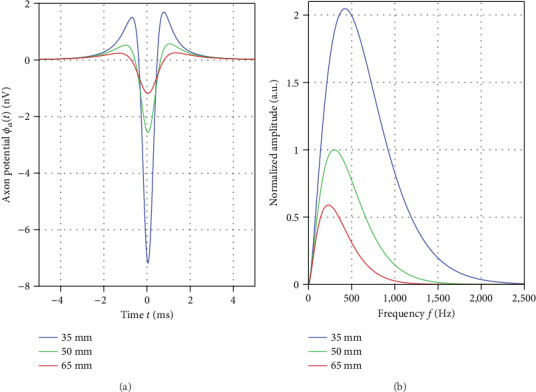
(a) Axonal signals *ϕ*_*A*_ for a conduction velocity of 60 ms^−1^. Color coding blue, green, and red correspond to a depth of 35 mm, 50 mm, and 65 mm, respectively. (b) Absolute amplitude obtained for the frequency spectrum of the three axonal signals. Color coding is identical as in the time domain. The spectral amplitude was normalized by the peak value obtained at *s* = 50 mm.

Figure 7 depicts the axonal potential *ϕ*_*A*_ at a fixed conduction velocity of 60 ms^−1^ for variable depths. As expected, axonal body surface amplitude was inversely proportional to approximately the square of the depth.

In our simulations, the volume conductor (lead field term) reduced the peak frequency of axons signals on the body surface. For the chosen parameter range, axonal peak frequency was between 200 Hz and 500 Hz. All axonal signals displayed spectra with a single peak as predicted by our model analysis. For this reason, both at low near DC frequencies and at high frequencies above several kHz, the amplitudes in the spectrum were very low. With increasing depth, the peak frequency decreased, in accordance with the prediction obtained from (8) for the estimated center frequency ${\tilde{f}}_{A}$.

*Dispersion.* The low-pass filter effect of temporal dispersion described in Section 2.3 further reduced the frequency content. For the CAP, peak frequency was in the range of 90 Hz to 170 Hz. The estimated center frequency ${\tilde{f}}_{N}$ was slightly above peak frequency and well within the computed frequency band. Thus, the frequency band was essentially defined by the inverse of the time span in which the majority of activation pulses pass the segment of $2\sqrt{2}s$ length. The peak-to-peak amplitude of the signal was in the order of magnitude of 1*μV*, which is a typical value for short latency somatosensory evoked potentials. Despite the assumption of 2000 axons defining a neural pathway, the CAP increased only by a factor of 200 compared to a single axon. This loss of amplitude was due to temporal dispersion, as individual axons generated their signals in an asynchronous fashion.

**Figure 8:**
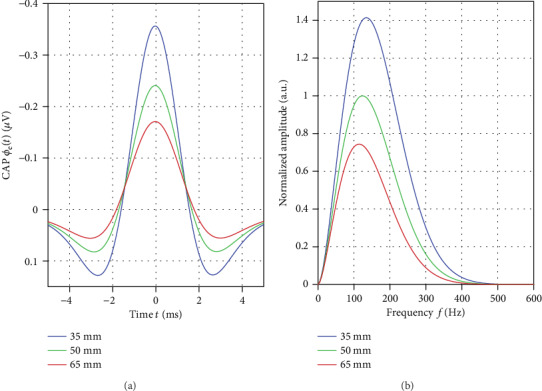
(a) Signals *ϕ*_*N*_ of the neural pathway for a conduction velocity of 60 ms^−1^. Negative amplitude was plotted upwards. Color coding is the same as in Figure 7. The time interval between the two positive peaks was in the range of 3 ms to 4 ms for all simulations. (b) Absolute amplitude obtained for the frequency spectrum of the three axonal signals. Amplitude was normalized by the peak value obtained at *s* = 50 mm.

Figure 8 depicts the potential of a neural pathway as a superposition of many axonal signals as defined in (4). Here, in accordance with clinical practice, negativity is plotted upwards. The width of the signal increased in relation to temporal dispersion in the volley. In the frequency spectrum, increased width was reflected by a shift of the maximum towards a lower frequency. The morphology of the signal again reflected the underlying quadrupole source pattern. Again, the amplitude was inversely related to the depth. However, the influence of depth was less than a power of two. This can be explained by the large spatial distribution of a volley along the axon.

Table 1 depicts results obtained with the middle range of assumed parameters (i.e., *s* = 50 mm, *v* = 60 ms^−1^, and *τ* = 1.25 ms). FT delivered peak frequencies and bandwidth (amplitude ≥ 50% of peak amplitude), which are listed together with the estimates $\tilde{f}$ (as defined in Section 2.4). The signals and their spectra are shown by the green traces in Figures 7 and 8.

Table 2 depicts the parameters for the highest, lowest, and middle peak frequencies in our simulation. For investigating the correlation of the peak frequency with the estimated center frequency $\tilde{f}$, we computed the coefficient of determination *r*^2^ from all parameter combinations. For the axonal signals, we obtained *r*_*A*_^2^ = 0.997, and for the CAPs, we obtained *r*_*N*_^2^ = 0.996. Thus, the estimated center frequencies $\tilde{f}$ as defined in Section 2.4 accurately reflected the effect of the parameters on the signal spectrum.

## 4. Discussion

With the increasing distance from a source to an observation electrode, spatial smoothing of the lead field reduced the high-frequency content in the axonal signal. The frequency band was in the range of several hundred Hz. This reduction was caused by the Laplace term which underlies each biopotential computation [23]. Note that this was a purely geometrical effect which was also described for body surface cardiac signals [10]. From our model analysis, we interpreted the obtained frequency band as follows: the observation electrode was most sensitive for signals in an axonal segment of $2\sqrt{2}s$ length underneath the electrode. The inverse of the time, which an activation pulse needed in order to pass this segment, was an estimator for the center of the frequency band. The peak-to-peak amplitude of a signal generated by a single axon on the body surface was in the order of nanovolts. Thus, our model predicted that individual activity of single axons cannot be observed from the body surface.

Any model is based on idealized assumptions and this is also true for an analytic model. In order to allow for an analytic treatment, we restricted our analysis to axons of equal diameter and a simplified half-space geometry. Thus, we limited our analysis in the first step to the traveling wave along the spinal cord—a waveform which is scientifically well described [15] but of restricted clinical use due to its low amplitude. The signals obtained from our model were in reasonable agreement with experimental data:

- CAP amplitudes were in the order of some tens of microvolts [15]. Here, the values chosen for conductivity and for the number of axons entered the result. There is considerable uncertainty in these parameters, and thus, only coarse estimates were obtained for the amplitude. The intracellular conductivity we used in our simulation was the inverse of the cytoplasmic resistivity as used in the cell model described in [14]. Compared to cell models, direct measurement of cytoplasmic resistivity yields similar values [24] when extrapolating data to body temperature [25]. The chosen bulk conductivity was an average value of different types of tissues which surround axons or nerves. Here, the selected conductivity ratio corresponds to a bulk conductivity of approximately 0.2 Sm^−1^ which is a frequently used value for average tissue conductivity [21]. We assumed that 2000 axons contributed to the signal generated by a spinal tract. As can be seen from Appendix C, we estimated that a neural structure containing 2000 axons had a diameter of 0.8 mm which appeared being a reasonable dimension. The total macaque pyramidal tract contains approximately 600000 axons [22]. We assumed a comparable number of axons in the sensory tracts, of which only a small portion is activated by stimulation of a peripheral nerve. Thus, our model allows for a coarse estimate of amplitude at reasonable tract dimensions. However, variation of parameters showed that there is still a huge uncertainty in amplitude estimations. Future work is needed for reducing this variability
- For single axons, the “quadrupole” signal morphology qualitatively agreed with early theoretical predictions [12] and recent experimental findings [26]. For the CAP, the dominating negative peak of activation was also in agreement with experimental findings [15].
- Our analytical framework predicted that the width of the temporal dispersion function is the main factor determining the width of the evoked CAP on the body surface. By selecting 4*τ* in the range of 4 ms to 6 ms, the width of the computed CAP was in the order of 5 ms to 10 ms which is comparable to experimental observations [15]. Here, peak or central frequencies were typically in the order of 100 Hz to 200 Hz. Literature on experimentally observed dispersion functions is largely lacking. Basic theory on characteristic functions of probability density functions predicts that the low-pass filter effect widening the signal trace will be observed for any dispersion function. Due to the limitations of the analytic model, we restricted our analysis to the normal distribution as a basic model of statistical distribution. As discussed below, numerical methods should be applied in future studies for addressing the temporal dispersion in combination with variable axon diameter and conduction velocity. In the first step, the chosen parameters allowed a reasonable prediction of the width of the CAPs and their central frequency band.

The simplifications described above did not allow using our model for studying signals generated by neurophysiologically relevant structures such as the spinal gray matter or subcortical signal generators [2, 15]. However, as there was some similarity between the signal morphology and width of lumbosacral stationary potentials with the CAP predicted by our models, our results might be also useful for a first estimation of spectral properties of lumbosacral postsynaptic EPs.

Our analysis on the 50% bandwidth of the signal suggested that most of the signal was contained in a relatively narrow band of 40 Hz to 300 Hz. Bandwidth was significantly more narrow than suggested in the guidelines (20 Hz to 3000 Hz [4]) and in agreement with current scientific work, which focuses on the range of several hundred hertz for neural high-frequency signals [5–8] rather than the kilohertz range. In a routine setting, a more strictly selected bandwidth might allow reducing the influence of technical noise sources such as thermal noise (electrode impedance and amplifier). Here, future experimental work is needed. With respect to physiological background activity, the CAP spectrum displayed a strong overlap with the activity of skeletal muscles and a weaker overlap with cardiac activity [27]. Signals generated by skeletal muscles are within a broad frequency band with significant spectral components in the range of 100 Hz to 400 Hz [28, 29]. Cardiac body surface potentials contain frequencies up to 150 Hz. However, the most significant ECG frequency components are at relatively low frequencies. For a standard 12-lead ECG, the bandwidth is normally reduced to 100 Hz, and for ECG monitoring in critical care units, the bandwidth is even reduced to 40 Hz [30].

## 5. Conclusions and Outlook

We developed a theoretical framework which allows the computation of lead field potentials generated by a neural tract, nerve, or fascicle beyond considering each single axon or fiber. Our approach allows linking membrane potentials obtained from ionic current models with body surface potentials for multiscale modeling. The neural structure was modeled by its central axis, and individual membrane action potential pulses were considered by their statistical distribution in time. Our results suggest that the signals generated by individual axons are sufficiently small and that the number of axons is sufficiently large for obtaining a reasonable approximation by statistical analysis.

Similarly, as for cardiac tissue [21, 31] or stimulation of cortical tissue [32], our model considers two domains: an active intracellular domain (membrane potential *V*, intracellular conductivity *σ*) and an extracellular bulk domain (potential *ϕ*, conductivity *κ*). In contrast to existing models, our approach focuses on lead field activity modeling the source field by dipole distributions. Then, the passive potential is obtained by integration. Here, convolution allows solving equations in the time and frequency domains.

The treatment in the frequency domain allowed gaining insight into the spectral properties of the generated signals. Highest frequencies occur directly at the cell membrane with peak frequencies in the order of kilohertz. The volume conductor transmitting the signal to a lead field electrode acts as a bandpass reducing frequency content with increasing distance between the source and sensor. Furthermore, temporal dispersion of individual activation pulses acts as an additional low-pass filter reducing peak or central frequency in the signal to approximately 100 Hz to 200 Hz. This suggests that the clinical bandwidth of 20 Hz to 3000 Hz [4] may be unnecessarily broad. Future experimental and modeling work is needed for potentially optimizing recommendations for bandwidth and filter types. This may significantly improve the clinical practicability of SEP recordings by reducing the number of samples needed for obtaining reliable recordings.

For establishing the concept of our theory, we applied analytic treatment. This limits analysis to a model of a simple half-space geometry. The results apply to the low-amplitude propagating wave in spinal evoked potentials.

The insight obtained from developing an analytical framework for a lead field two-domain model of a neural tract or nerve provides a sound basis for using numerical tools in the near future for generalizing the approach. This will allow the investigation of a broader spectrum of anatomically and electrophysiologically realistic modeling applications:

- Numerical field computation schemes such as the finite element method (FEM) and the boundary element method (BEM) [33–35] can be used for computing the lead field matrix generated by a unit dipole traveling along a central axis of a neural pathway. Such methods allow considering realistic anatomy and variation of conductivity in tissue compartments. By convolution of such a numerically computed lead field matrix with the primary source matrix the axonal potential can be computed in general geometries. For example, such methods can be used for computing the potential generated by an axon of a peripheral nerve in a limb.
- Our approach allows numerical computation of axonal signals for fibers of varying diameter (and thus, varying conduction velocity). The model can be generalized to neural tracts containing fibers of varying diameter, by applying numerical tools for summation of many axonal signals (instead of statistical methods as used here). This will allow predicting dispersion at the stimulation site of a peripheral nerve applying computer modeling as described in [13, 14]. By considering individual conduction properties of fibers along the nerve, the lead field two-domain model can be generalized for computing CAPs of peripheral nerves or spinal tracts.
- Dispersion functions may be a powerful tool for computing also the lead field potentials of stationary signal generators by direct convolution of a membrane potential derivative with a convolution function.

Thus, the lead field two-domain model has the potential for providing an important novel model-based research tool for evoked or stimulated neurophysiological signals.

## Figures and Tables

**Table 1:** Results for parameter setting *s* = 50 mm, *v* = 60 ms^−1^, and *τ* = 1.25 ms.

	Peak frequency	Bandwidth	Frequency estimates	Amplitude
	(Hz)	(Hz)	(Hz)	(peak-to-peak)
AP derivative, *dV*/*dt*	1830	547 to 4545	1750	3.1 *V*ms^−1^
Axon potential, *φ_A_*	300	100 to 661	338	1.24 n*V*
CAP, *φ_N_*	125	50 to 227	126	0.32 *μV*

**Table 2:** Simulation parameters and characteristic frequencies.

Depth	Conduction velocity	Peak frequency	Bandwidth	Frequency estimates
(mm)	(ms^−1^)	(Hz)	(Hz)	(Hz)
35	75	170	69 to 302	169
50	60	125	50 to 227	126
65	45	90	36 to 173	94

## Data Availability

The data contained in the article were obtained from simulations which were encoded in Matlab based on the analytical derivations described in the article. All data used to support the findings of this study are available from the corresponding author upon request.

## Supplementary Materials

Supplementary Materials The supplementary file is named R1_Appendix_CompMathMethMed.
