# Supplementary material for: A Lead Field Two-Domain Model for Longitudinal Neural Tracts—Analytical Framework and Implications for Signal Bandwidth
Source: Comput Math Methods Med. 2020 May 29;2020:5436807. doi: 10.1155/2020/5436807 (PMC7275970; doi:10.1155/2020/5436807)
Supplement: Supplementary Materials — The supplementary file is named R1_Appendix_CompMathMethMed. [file 5436807.f1.pdf]

Online Supplement for:

A Lead Field ~~Bid~~Two-Domain Model for Longitudinal Neural Tracts – Analytical Framework and Implications for Signal Bandwidth

Gerald Fischer, Markus Kofler, Michael Handler, Daniel Baumgarten

March 2020

This documents provides the Appendix for the manuscript submitted to Computational and Mathematical Methods in Medicine.

Appendices

A Axonal Dipoles Model

A.1 Analytical Treatment

The potential ϕ generated by a dipole p in a infinite conducting space of conductivity κ in defined by (see [3]; chapter 8; eq. (8.15)):

$$\phi(r) = \frac{p\cos(\theta)}{4\pi\kappa r^2}. \tag{1}$$

Here, r is the distance between the source point (dipole) and the field point (electrode e) and θ is the angle between the orientation vector of the dipole and the source point to field point vector.

For modeling a half space geometry, we fix the observation electrode e at the origin and allow the dipole to translate at $y = 0, z = -s$ along the x coordinate. The non-conducting boundary of the half space ($z = 0$) imposes a Neumann boundary condition. The Neumann boundary is considered by mirroring the dipole relative to the boundary (see [5], chapter 2.3 and figure A.1). We obtain:

$$\phi = -\frac{p}{2\pi\kappa}\frac{x}{(s^2+x^2)^{\frac{3}{2}}}. \tag{2}$$

Equation (2) describes the field ϕ a source p generates at the electrode e. Thus, it describes the lead field.

We can model the impressed current density along the intracellular space of the axon by a distribution of dipoles dp of infinitesimal strength. The strength of the dipoles is defined by the impressed intracellular currents driven by the gradient of the membrane potential V:

$$\mathrm{d}p = -\frac{dV}{dx}\frac{\pi}{4}d^2\sigma\,\mathrm{d}x \tag{3}$$

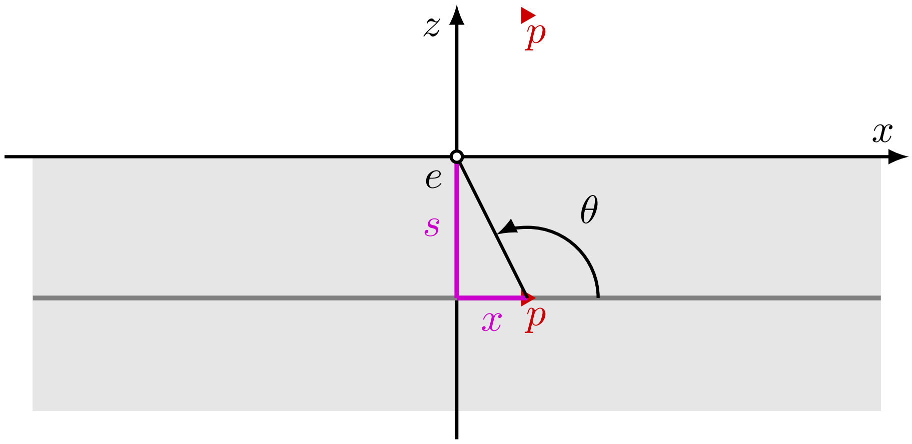

Figure A.1: The dipole p is placed in a conducting half space at a depth s and shifted along the axon by the coordinate x. For considering the nonconducting boundary the dipole is mirrored at $z = 0$.

Here σ is the conductivity of the intracellular space and d the diameter of the axon.

We assume that the action potential propagates undistorted with a velocity v along the axon. Thus, at the coordinate x the action potential is shifted in time by an offset T:

$$V(x=0,t) = V(x, t-T) \ldots T = t - \frac{x}{v}. \tag{4}$$

We can substitute $x = vt$:

$$\frac{\mathrm{d}V(x)}{\mathrm{d}x} = -\frac{\mathrm{d}V(t-\frac{x}{v})}{\mathrm{d}(vt)} = -\frac{1}{v}\frac{\mathrm{d}V(t-\frac{x}{v})}{\mathrm{d}t}. \tag{5}$$

Thus, we obtain for the distribution of dipoles dp:

$$\mathrm{d}p = \frac{\pi d^2 \sigma}{4v}\frac{\mathrm{d}V(t-\frac{x}{v})}{\mathrm{d}t}\,\mathrm{d}x\,. \tag{6}$$

By substituting (6) into the lead field (2) we obtain the potential which is produced by an infinitely small dipole dp at the electrode e:

$$\mathrm{d}\phi_A = -\frac{d^2}{2v}\frac{\sigma}{\kappa}\frac{\mathrm{d}V(t-\frac{x}{v})}{\mathrm{d}t}\frac{x}{(s^2+x^2)^{\frac{3}{2}}}\,\mathrm{d}x\,. \tag{7}$$

We obtain the axonal potential ϕ_A by integrating the contributions of all dipoles along the x coordinate:

$$\phi_A(t) = -\frac{d^2}{8v}\frac{\sigma}{\kappa}\int_{-\infty}^{\infty}\frac{\mathrm{d}V(t-\frac{x}{v})}{\mathrm{d}t}\frac{x}{(s^2+x^2)^{\frac{3}{2}}}\,\mathrm{d}x\,. \tag{8}$$

We substitute $x = v\tau$ for performing integration in the time domain:

$$\phi_A(t) = -\frac{d^2}{8}\frac{\sigma}{\kappa}\int_{-\infty}^{\infty}\frac{\mathrm{d}V(t-\tau)}{\mathrm{d}t}\frac{v\tau}{(s^2+v^2\tau^2)^{\frac{3}{2}}}\,\mathrm{d}\tau\,. \tag{9}$$

We observe that the axonal potential ϕ_A is obtained from a convolution of the action potential derivative with the lead field.

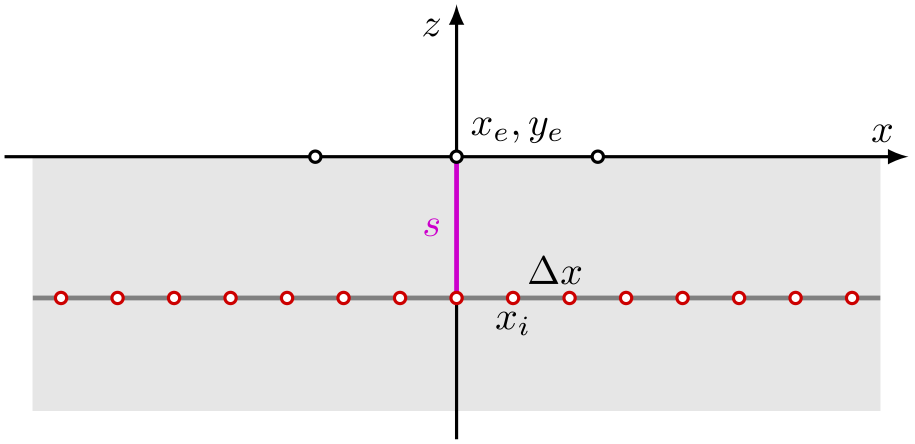

Figure A.2: Schematic drawing of the discrete model underlying the numerical approximation. At the body surface the potential is calculated in E electrodes (black circles, coordinates x_e, y_e). The axon is subdivided into I source points x_i at spacing Δx (red circles).

We performed our analysis above for an electrode located at the origin (i.e. $x_e = y_e = 0$). We can extend our analysis to any body surface location by condering general electrode coordinates x_e, y_e. We than can rewrite (2) by using (1):

$$\phi(x_e, y_e) = -\frac{p}{2\pi\kappa} \frac{x - x_e}{(s^2 + (x - x_e)^2 + y_e^2)^{\frac{3}{2}}}. \tag{10}$$

We can now repeat the calculation above for a general electrode location x_e, y_e and obtain for the axonal potential $\phi_A(x_e, y_e, t)$:

$$\phi_A(x_e, y_e, t) = -\frac{d^2}{8}\frac{\sigma}{\kappa} \int_{-\infty}^{\infty} \frac{\mathrm{d}V(t-\tau)}{\mathrm{d}t} \frac{v\tau - x_e}{[s^2 + (v\tau - x_e)^2 + y_e^2]^{\frac{3}{2}}} \mathrm{d}\tau\,. \tag{11}$$

A.2 Numerical Approximation

In this subsection we show that the axonal potential ϕ_A in E electrodes x_e can be approximated by a linear equation. For the scope of numerical approximation the axon is represented by I discrete source points x_i evenly spaced by Δx along the finite length of the discrete axon model (Figure A.2). For computing the potential at a single time step t_m we can replace the integral in (11) by a discrete summation over all source points

$$\phi_A(x_e, y_e, t_m) \approx -\frac{d^2}{8v}\frac{\sigma}{\kappa} \sum_I \frac{x_i - x_e}{[s^2 + (x_i - x_e)^2 + y_e^2]^{\frac{3}{2}}} \frac{\mathrm{d}V(t_m - \frac{x_i}{v})}{\mathrm{d}t} \Delta x. \tag{12}$$

We have replaced $v\tau$ by x_i and the time step Δt by $\frac{\Delta x}{v}$. For E electrodes at M time steps we can rewrite equation (12) by a linear system

$$\mathbf{\Phi}_A = \mathbf{LD}. \tag{13}$$

Here $\mathbf{L}$ is the lead field matrix relating the source points to the leads. Its elements $l(e, i)$ are defined by

$$l(e,i) = \frac{x_i - x_e}{[s^2 + (x_i - x_e)^2 + y_e^2]^{\frac{3}{2}}}, \tag{14}$$

and $\mathbf{D}$ is the dipole matrix. It elements $\delta(s,m)$ assign a source strength to each dipole location at each time step t_m

$$\delta(s,m) = -\frac{d^2}{8v}\frac{\sigma}{\kappa}\frac{\mathrm{d}V(t_m - \frac{x_i}{v})}{\mathrm{d}t}\Delta x. \tag{15}$$

The matrix $\mathbf{\Phi}_A$ holds the axon potentials at M time steps in E electrodes.

B Dispersion Model

B.1 General Distribution –Harmonic function:

We assume that the signal of a neural structure is composed from the activity of N axons which are dispersed by a temporal offset or time shift T. The statistic distribution of the time shift can be described by a dispersion or distribution function $\delta(T)$. The following properties of a statistic distribution function $\delta(T)$ follow from basic theory. The dispersion $\delta(T)$ does not contain any negative values. Thus, we can write:

$$\delta(T) \geq 0 \ \ldots \ \forall T \in \mathbb{R}. \tag{16}$$

Furthermore, the area of the distribution function $\delta(T)$ equals one:

$$\int_{-\infty}^{\infty} \delta(T)\,\mathrm{d}T = 1. \tag{17}$$

In addition, the dispersion $\delta(T)$ is piece-wise continuous.

The CAP signal $\phi_N(t)$ generated by N axons in a neural tract is generated by the sum of all individual axon potentials ϕ_A. Approximating this sum by the integral over the dispersion function $\delta(T)$ we can write in the time domain:

$$\phi_N(t) = N\int_{-\infty}^{\infty} \delta(T)\phi_A(t-T)\,\mathrm{d}T\,. \tag{18}$$

We observe, that ϕ_A is obtained from a convolution of the axonal signal ϕ_A with the disperstion $\delta(T)$. Thus, we obtain in the time domain:

$$\mathscr{F}\{\phi_N(t)\} = 2\pi N \mathscr{F}\{\phi_A(t)\}\mathscr{F}\{\delta(t)\}. \tag{19}$$

We observe that the spectrum of the CAP signal $\mathscr{F}\{\phi_N(t)\}$ is obtained from the product of the spectrum of the axonal signal $\mathscr{F}\{\phi_A(t)\}$ and the spectrum of the distribution function $\mathscr{F}\{\delta(t)\}$.

The spectrum of a distribution function $\mathscr{F}\{\delta(t)\}$ is called its harmonic function $\Phi(\omega)$ [2]. Due to the basic properties of distribution functions as described above, also the harmonic function is characterized by basic properties. We summarize some of them, which are important for our analysis.

As the area under the distribution function $\delta(T)$ yields one, it follows that the DC-content $\Phi(0)$ is one [2]:

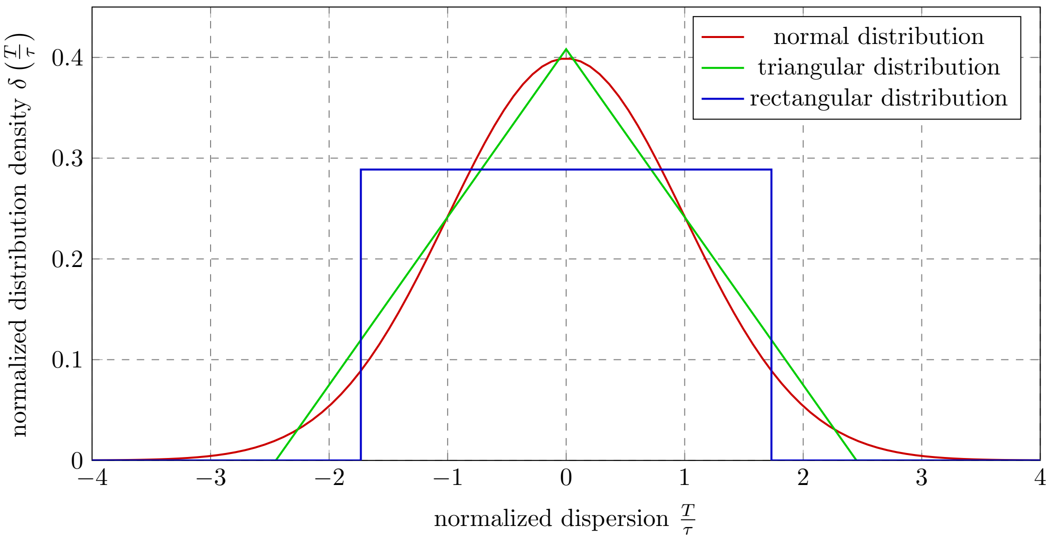

Figure B.1: Three example distribution functions

$$\Phi(\omega = 0) = 1. \tag{20}$$

Furthermore, it follows from (16) and (17) the maximal amplitude of a harmonic function $\Phi(\omega)$ is one:

$$|\Phi(\omega)| \leq 1 \ \ldots \ \forall \omega \in \mathbb{R}. \tag{21}$$

In addition, $\Phi(\omega)$ is a continuous function [2]. Finally, as $\delta(T)$ is piece-wise continuous the harmonic function converges to zero as ω approaches infinity:

$$\lim_{\omega \to \pm\infty} \Phi(\omega) \to 0. \tag{22}$$

From equations (20) to (22) it follows that the spectrum of an harmonic function has the characteristic of a low pass filter. We obtain the maximal amplitude at $\omega = 0$ and we obtain a decreasing amplitude when frequency increases to high values.

B.2 Example Distributions

In this ~~chapter~~section we compute the harmonic function of some example distributions for illustrating the validity of the general treatment described above. For quantitatively comparing distribution functions of different shape we define parameters such that all investigated distribution function have the identical standard deviation τ.

Normal Distribution

We assume that dispersion of activation in N axons can be approximated by a normal distribution $\delta_n(T)$ of standard deviation τ. We can write:

$$\delta_n(T) = \frac{1}{\sqrt{2\pi}\tau} \exp\left(-\frac{T^2}{2\tau^2}\right). \tag{23}$$

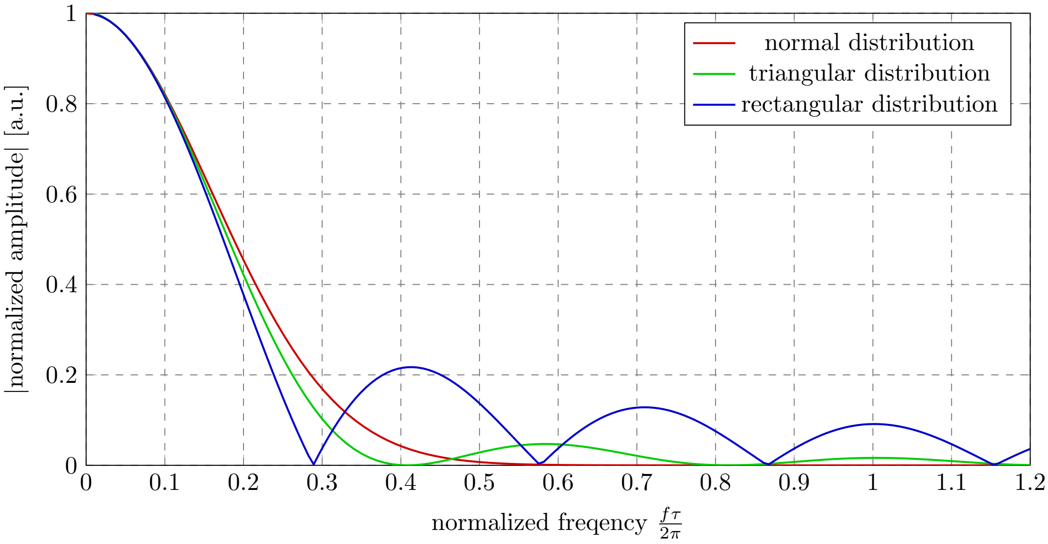

Figure B.2: Harmonic functions of the three example distribution functions. Note that they all fullfill the basic properties as described in equations (20) to (22).

The distribution function $\delta_n(T)$ is shown in Figure B.2. We obtain the CAP spectrum using (19). According to [2] characteristic function of $\delta_n(T)$ is:

$$\mathscr{F}\{\delta(T)\} = \exp\left(-\frac{\tau^2\omega^2}{2}\right). \tag{24}$$

Triangular Distribution

We consider a triangular distribution $\delta_t(T)$ of width $\pm T_t$:

$$\delta_t(T) = \begin{cases} \frac{1}{T_t}\left(1 - \frac{|T|}{T_t}\right) & \text{for} - T_t \leq T \leq T_t, \\ 0 & \text{else}. \end{cases} \tag{25}$$

Note that the area under the distribution function yields one. We obtain from basic calculus of time continuous FT:

$$\mathscr{F}\{\delta_t(T)\} = \sqrt{\frac{2}{\pi}}\frac{N}{\omega^2 T_t^2}[1 - \cos(\omega T_t)]. \tag{26}$$

For enabling a direct comparison of distributions we need to relate T_t with τ. We can write (see [6], page 109, equation (6.28)):

$$\tau^2 = \frac{2}{T_t}\int_0^{T_t} T^2\left(1 - \frac{T}{T_r}\right)\mathrm{d}T\,. \tag{27}$$

We obtain by solving (27) for T_t:

$$T_r = \sqrt{6}\tau. \tag{28}$$

Thus, by inserting (28) into (26) we can compute the harmonic function of a triangular distribution for a given standard deviation τ.

Rectangular Distribution

We consider a rectangular distribution $\delta_r(T)$ of width $\pm T_r$:

$$\delta_r(T) = \begin{cases} \frac{1}{2T_r} & \text{for} - T_r \leq T \leq T_r, \\ 0 & \text{else.} \end{cases} \tag{29}$$

Note that the area under the distribution function yields one. We obtain from basic calculus of time continuous FT:

$$\mathscr{F}\{\delta_r(T)\} = \sqrt{\frac{2}{\pi}} \frac{\sin(\omega T_r)}{\omega}. \tag{30}$$

For enabling a direct comparison of distributions we need to relate T_r to τ. We can write:

$$\tau^2 = \frac{1}{T_r} \int_0^{T_r} T^2 \, \mathrm{d}T \, . \tag{31}$$

We obtain by solving (31) for T_r:

$$T_r = \sqrt{3}\tau. \tag{32}$$

Thus, by inserting (32) into (30) we can compute the harmonic function of a rectangular distribution at a known standard deviation.

C Cross-section of Neural Tract

We aim to estimate the fraction ν of the cross-sectional area of a nerve which is occupied by the intracellualar space. The upper bound for ν can by obtained from the axon arrangement shown in figure C.1. Here, all axons have identical dimensions and a circular cross-section. Thus, they are as densely packed as possible. We write d for the diameter of the intracellular space and w for the width of the myelin layer.

We obtain the upper bound for ν by the fraction of the area A_I of three intracellular compartments contained in the triangular portion in figure C.1 with the entire area of the triangle A_T. The triangle area A_T is defined by:

$$A_T = (d + 2w)^2 \frac{\sqrt{3}}{4}. \tag{33}$$

The three intracellular compartments together yield half of the cross-section of a circle with a diameter d. We obtain:

$$A_I = d^2 \frac{\pi}{8}. \tag{34}$$

Thus, the upper bound of ν is obtained from:

$$\nu \leq \frac{A_I}{A_T} = \frac{\pi}{2\sqrt{3}} \frac{1}{\left(1 + 2\frac{w}{d}\right)^2}. \tag{35}$$

Experimental data for the fraction of w with d can be obtained from the studies by [1, 4]. The both report consistently an approximate value of $\frac{w}{d} \approx 0.2$. Thus, we obtain:

$$\frac{A_I}{A_T} \approx 0.46. \tag{36}$$

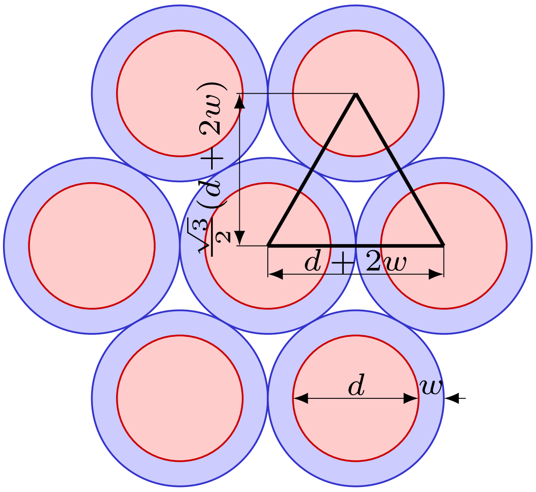

Figure C.1: Densely packed axons of circular geometry and equal dimensions. The intracellular space of diameter d is depicted in a red shading. The myelin layer of a width w is depicted is a blue shading. A triangular area is investigated for calculating surface ratios (see text).

Due to the unequal size and shape of axons a realistic value is somewhat below the upper bound estimation. It appears reasonable that approximately one third of the cross-section of a neural tract is occupied by intra-cellular space, yielding $\nu \approx 0.33$.

$$D = \sqrt{\frac{Nd^2}{\nu}}. \tag{37}$$

Thus, for a neural tract containing $N = 2000$ fibers of diameter $d = 10\,\mu\text{m}$, we obtain an outer diameter of $D = 0.78\,\text{mm}$.

References

[1] M. J. Gillespie and R. B. Stein. The relationship between axon diameter, myelin thickness and conduction velocity during atrophy of mammalian peripheral nerves. *Brain Res.*, 259(1):41–56, Jan 1983.

[2] E. Jondeau. Characteristic functions and fourier transforms. In E. Jondeau, S.H. Poon, and M. Rockinger, editors, *Financial Modeling Under Non-Gaussian Distributions*, pages 477–486. Springer London, London, 2007.

[3] J. Malmivuo and R. Plonsey. *Bioelectromagnetism - Principles and Applications of Bioelectric and Biomagnetic Fields*. Oxford University Press, New York, 1995.

[4] J. M. Schroder, J. Bohl, and U. von Bardeleben. Changes of the ratio between myelin thickness and axon diameter in human developing sural, femoral, ulnar, facial, and trochlear nerves. *Acta Neuropathol.*, 76(5):471–483, 1988.

[5] K. Simonyi. *Theoretische Elektrotechnik*. Barth, 1993.

[6] C. Weiß and B. Rzany. *Basiswissen Medizinische Statistik*. Springer-Lehrbuch. Springer Berlin Heidelberg, 2013.
